# Long-term outcome of thyroid lobectomy for unilateral multifocal papillary carcinoma

**DOI:** 10.1097/MD.0000000000007461

**Published:** 2017-07-07

**Authors:** Hui Huang, Shaoyan Liu, Zhengang Xu, Song Ni, Zongmin Zhang, Xiaolei Wang

**Affiliations:** Department of Head and Neck Surgery, National Cancer Center /Cancer Hospital, Chinese Academy of Medical Sciences, Peking Union Medical College, Beijing, China.

**Keywords:** multifocality, papillary thyroid carcinoma, prognosis, surgical treatment

## Abstract

The National Comprehensive Cancer Network (NCCN) guidelines recommend completion thyroidectomy for patients with multifocal papillary thyroid carcinoma (PTC) diagnosed by paraffin pathology after lobectomy. However, studies for the influence of surgical range on prognosis of unilateral multifocal carcinoma are scarce. We analyzed the clinicopathological characteristics and long-term outcomes of patients with unilateral multifocal PTC to identify risk factors for recurrence and disease-related death.

The clinical and pathological data of 123 cases with multifocal lesions in the unilateral thyroid lobe were retrospectively collected, including sex, age, stage, surgical range, histopathology characteristics, and follow-up data. The prognostic factors were analyzed by means of the Kaplan–Meier method.

The recurrence in the contralateral residual thyroid was observed in 6 cases. The 10-year cumulative recurrence rate of the remnant thyroid was 7.0%. Extrathyroidal extension (ETE) was a significant prognostic factor, with χ^2^ equal to 4.043 and a *P* value of .044. One patient died from progression of pulmonary metastasis during the follow-up. The 10-year disease-specific survival rate was 96% and 14 cases experienced recurrences and underwent a second surgery (11.4%), and thus the 10-year recurrence-free survival rate was 83.2%. Multivariate analysis showed that the pathologic tumor (pT) stage was an independent prognostic factor for the recurrence-free survival rate (*P* <.0001, hazard ratio 2.871, 95% confidence interval 1.783–4.624).

ETE is a significant prognostic factor for the recurrence of the remnant thyroid and pT stage is an independent prognostic factor for tumor recurrence-free survival. Lobectomy (with isthmectomy) is effective for most patients with unilateral multifocal PTC.

## 1 Introduction

Papillary thyroid carcinoma (PTC) is a type of indolent tumor with a good prognosis. The biological behaviors of multifocal PTC are significantly worse than those of solitary carcinoma. The incidence of cervical lymph node metastasis and distant metastasis is significantly higher than that of solitary carcinoma, and the incidence of extrathyroidal extension (ETE) or T stage is significantly higher than that of solitary carcinoma; multifocality is a risk factor for recurrence and a poor prognosis.^[1–5]^ Therefore, bilateral total thyroidectomy is a widely recognized surgical option for multifocal PTC.^[6,7]^

However, due to the limitations of B ultrasound examination and intraoperative frozen pathology, some cases of multifocal PTC are diagnosed by postoperative paraffin pathology. The National Comprehensive Cancer Network (NCCN) guidelines recommend completion thyroidectomy for patients with multifocal PTC diagnosed by surgical pathology.^[6]^

The current guidelines are mostly based on cross-sectional studies that suggest that unilateral multifocal carcinoma is an independent predictive factor of the presence of contralateral occult carcinoma.^[2,8–13]^ However, studies for the influence of surgical range on prognosis of unilateral multifocal carcinoma are scarce.^[9,10]^

In the past, the treatment principle of unilateral lesions in our institute was resection of the affected lobe, and patients who were diagnosed as having unilateral multifocal lesions after lobectomy did not undergo completion thyroidectomy. This study retrospectively analyzed the clinicopathological characteristics and long-term outcomes of patients with unilateral multifocal PTC to identify risk factors for recurrence and disease-related death.

## 2 Materials and methods

### 2.1 Research design

A total of 1601 cases of patients with papillary thyroid carcinoma who underwent primary treatment in our institute were retrospectively collected from January 1999 to December 2008, including 470 cases with multifocal lesions (29.4%) and 136 cases with multifocal lesions in the unilateral thyroid lobe (8.5%). All patients underwent routine preoperative ultrasonography of the thyroid and neck and chest x-ray film examination. Enhanced computed tomography scan of the neck and upper mediastinum was performed for the patients with apparent lateral cervical lymph node metastasis. Among them, 7 cases showed incomplete data, including preoperative examination (ultrasonography), surgical records, and pathological results, and 6 cases underwent total thyroidectomy. The pathological and clinical data of 123 cases with multifocal PTC in the unilateral thyroid lobe and complete data of patients who did not undergo total thyroidectomy were retrospectively collected, including sex, age, stage, surgical approach, histopathology characteristics, and follow-up data. The prognostic factors were analyzed. Staging was performed according to the American Joint Committee on Cancer TNM Stage for Thyroid Cancer (the 7th Edition, 2010). This is a retrospective analysis and would not cause any harm to patients, informed consent was not taken from the patients, but was approved by the institutional ethics committee.

### 2.2 General information

Thirty-nine cases were men and 84 cases were women, with a median age of 43 years (15–78). Among them, 74 cases were <45 years old and 49 cases were ≥45 years old. The routine preoperative ultrasonography examination showed that nodules were located in the unilateral thyroid lobe in 83 cases. And benign nodules were found in the contralateral glandular lobe in 40 cases. Preoperative assessment showed lateral cervical lymph node metastasis (cN1b) in 32 cases. Preoperative indirect laryngoscopy examination showed vocal cord paralysis in the affected side in 3 cases (see Table 1).

**Table 1:**
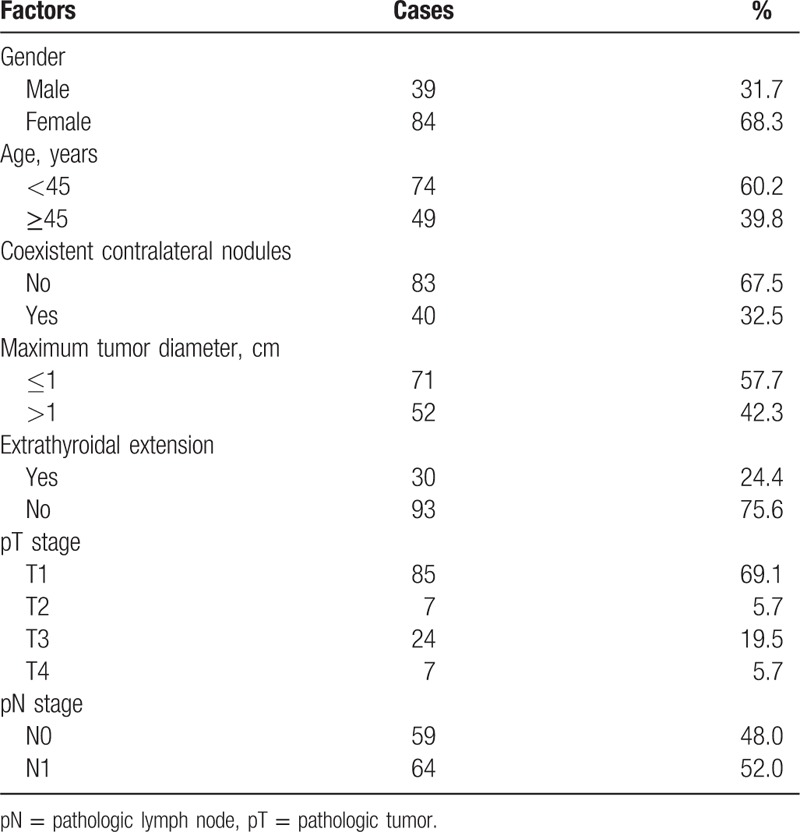
Clinicopathological characteristics.

### 2.3 Treatment

The principle of primary surgery was resection of at least the affected lobe and isthmus. Palpable nodules in the contralateral lobe were also resected during the surgery, including 82 cases with resection of the ipsilateral lobe and isthmus, 34 cases with resection of the ipsilateral lobe, isthmus, and partial contralateral lobe (or nodules), and 7 cases with resection of the ipsilateral lobe, isthmus, and subtotal contralateral lobe. Central lymph node dissection was performed in 67 cases (for patients with suspicious lymphadenopathy during the surgery or with lateral cervical lymph node metastasis, including bilateral dissection in 3 cases). Lateral cervical lymph node dissection was performed in 39 cases. Except for 8 patients who were lost to follow-up, all patients were treated with levothyroxine tablets and none of them were treated with iodine 131.

### 2.4 Follow-up and statistical analysis

The starting time of follow-up was the date of surgery and patients were followed up until November 15, 2016. The primary end points were recurrence rate in the remnant thyroid and recurrence-free survival. The follow-up data were obtained from electronic records of medical records or by mails/telephone calls. Eight cases (6.5%) were lost. The median follow-up time was 96 months (12–157). SPSS v 20.0 software was used for statistical analysis. Survival data were analyzed by means of the Kaplan–Meier method and survival curves were compared using the log-rank test in univariate analysis. And Cox regression analysis was used for multivariate analysis. All statistical tests were 2-sided and *P* values less than .05 were defined as statistically significant.

## 3 Results

### 3.1 Pathological characteristics

All 123 cases were diagnosed as unilateral multifocal PTC by paraffin pathology, including 71 cases with a maximum diameter ≤10 mm and 52 cases with a maximum diameter >10 mm. No thyroid capsular invasion was observed in 45 cases, capsular invasion but no extra thyroid extension was observed in 48 cases, and ETE was observed in 30 cases. There were pT1: 85 cases, pT2: 7 cases, pT3: 24 cases, and pT4: 7 cases. Central lymph node metastasis was observed in 53 cases (including 3 cases with bilateral metastasis) and lateral cervical lymph node metastasis was observed in 41 cases (including 2 cases negative by an intraoperative frozen biopsy but confirmed with metastasis by paraffin pathology). The total rate of lymph node metastasis was 52%, and the metastasis rate of lateral cervical lymph nodes was 33.3% (Table 1).

### 3.2 Recurrence and survival

One patient died from progression of pulmonary metastasis during the follow-up. The 10-year disease specific survival rate was 96%. Fourteen cases experienced recurrences and underwent a second surgery (11.4%), and thus the 10-year recurrence-free survival rate was 83.2%. The maximum diameter of the tumor, ETE, the pathologic tumor (pT) stage, and the lymph node status were significant prognostic factors (for details see Table 2). Multivariate analysis showed that the pT stage was an independent prognostic factor (*P *<.0001, hazard ratio (HR) 2.871, 95% confidence interval (CI) 1.783–4.624).

**Table 2:**
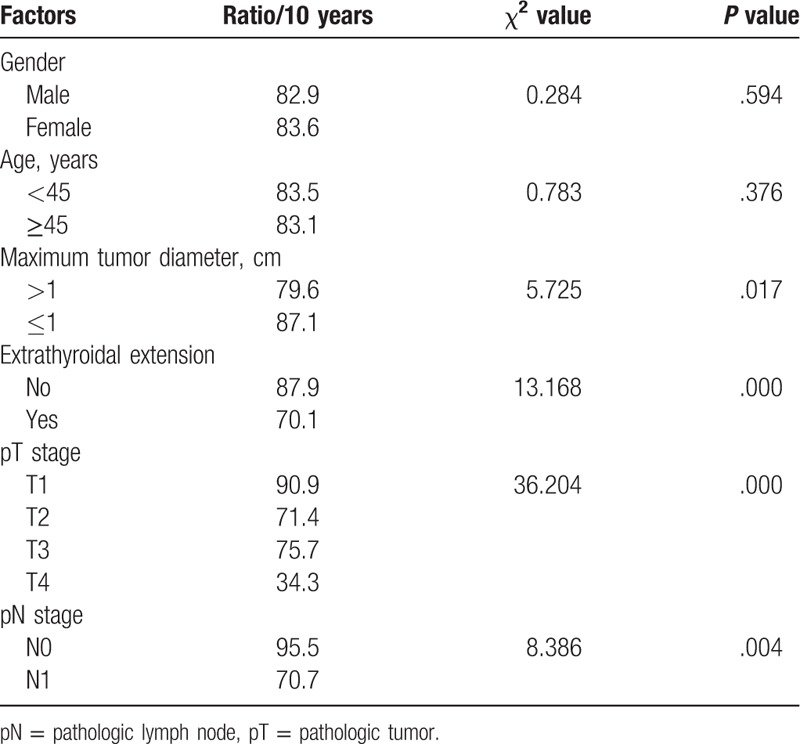
Univariate analysis of prognostic factors for recurrence-free survival.

The recurrence in the contralateral residual thyroid was observed in 6 cases, accounting for 4.88% of all patients (6/123). The 10-year cumulative recurrence rate of the remnant thyroid was 7.0%, 13.2% in cases with ETE, and 5.2% in cases with no ETE. ETE was a significant prognostic factor, with χ^2^ equal to 4.043 and a *P* value of .044. The 10-year cumulative recurrence rate in the remnant thyroid of patients with nodules in the contralateral lobe was 5.6%, and was 7.8% for patients without a nodule in the contralateral lobe (χ^2^ was 0.04, and the *P* value was .907) (for details see Table 3). Multivariate analysis showed no significant difference. Remnant thyroid resection was also performed in 6 cases for the cervical and/or superior mediastinal lymph node recurrence and in 1 case for pulmonary metastasis, and postoperative pathology showed no tumor in the remnant thyroid in all the 7 cases. Thirteen patients (10.56%) underwent a second surgery resecting the remnant thyroid.

**Table 3:**
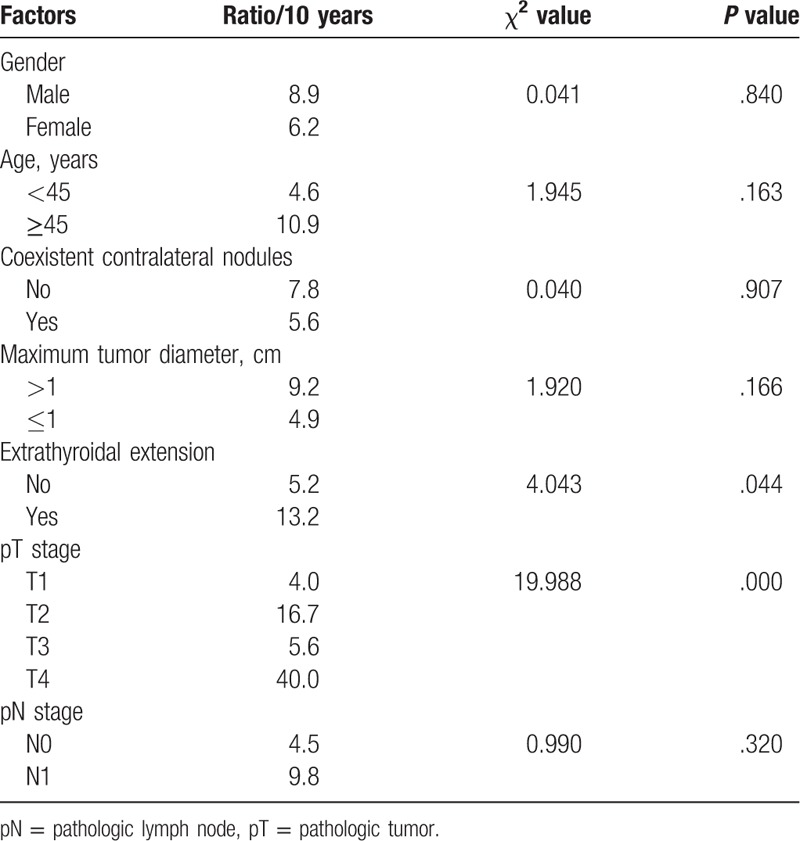
Univariate analysis of prognostic factors for recurrence rate of remnant thyroid.

During the same period, 934 patients diagnosed with unifocal papillary thyroid carcinoma were treated with lobectomy (with isthmectomy). The median follow-up time was 95 months. The 10-year cumulative recurrence rate of remnant thyroid was 8.7%, with no significant difference compared with unilateral multifocal cases (*P* = .866) (details not presented in this paper).

## 4 Discussion

PTC accounts for more than 90% of all thyroid malignant tumors, and multifocal PTC accounts for 17% to 45% of cases.^[1,2,8–12]^ This rate varies across reports, which may be due to population differences and small sample sizes. In the analysis of consecutive cases in our institute from 1999 to 2008, multifocal PTC was observed in 28.6% of cases. Although PTC is an indolent cancer, the biological behaviors of multifocal PTC are significantly worse than those of solitary PTC, with a higher rate of cervical lymph node metastasis, distant metastasis, and ETE.^[1–5]^

The most common recommendation for multifocal PTC is bilateral total thyroidectomy.^[6,7]^ Studies have shown that unilateral multifocality of the primary tumor and the presence of a coexistent benign nodule in the contralateral lobe by preoperative evaluation were independent predictive factors for occult contralateral PTC.^[8,9,11,12]^ Wu et al^[11]^ performed total thyroidectomy in 347 patients with unilateral papillary microcarcinoma combined with coexistent nodules in the contralateral lobe, and occult contralateral PTC was observed in 28.9%. Multivariate analysis showed that unilateral multifocality, presence of coexistent benign nodules in the contralateral lobe, and maximum tumor diameter greater than 5 mm were independent predictive factors of contralateral occult carcinoma, total thyroidectomy was thus suggested for patients with unilateral papillary microcarcinoma if they had any of the following factors: maximum tumor diameter greater than 5 mm, unilateral multifocality, or the presence of coexistent nodules in the contralateral lobe. NCCN guidelines state that multifocality is an indication for total thyroidectomy, and if unilateral multifocal carcinoma is pathologically diagnosed after thyroid lobectomy, completion thyroidectomy is also recommended.^[6]^

As unilateral multifocal carcinoma and the presence of a coexistent benign nodule in the contralateral lobe are predictive factors of contralateral occult carcinoma, total thyroidectomy is recommended for unilateral multifocal carcinoma.^[1,11–13]^ However, it is unreasonable to perform total thyroidectomy in all patients when only a few may have contralateral occult carcinoma and therefore most patients might become victims of over-treatment. Moreover, the incidence of hypoparathyroidism and recurrent laryngeal nerve injury are higher after total thyroidectomy than lobectomy. A large cohort study in the United States showed that the incidence of parathyroid injury after total thyroidectomy was 12.4% to 14.2%, and even surgeons who were technically mature and had a high volume of operations could not completely avoid injury to the recurrent laryngeal nerve.^[14]^

There is no reliable evidence demonstrating the effects of surgery range of unilateral multifocal carcinoma on long-term survival. Turanli et al^[9]^ found that 20.6% of patients with unilateral carcinoma also had contralateral occult carcinoma and unilateral multifocality was an independent predictive factor of occult carcinoma. However, contralateral occult carcinoma had no effect on survival and thus the authors suggested that total thyroidectomy was not mandatory for all patients. Grigsby et al^[10]^ performed completion thyroidectomy in all cases of PTC after lobectomy, and 41% had PTC in the contralateral lobe. There were no recurrence or survival differences in patients with or without contralateral disease after resection and radioactive iodine treatment.

In our institute, most patients with unilateral PTC were treated with lobectomy (with isthmectomy), and the patients diagnosed with unilateral multifocal carcinoma after lobectomy did not receive completion thyroidectomy but were observed during follow-up. The cumulative recurrence rate of remnant thyroid in this study was only 7%, which is lower than the prevalence of contralateral occult carcinoma reported in the literature (17%–45%). In other words, the majority of occult carcinoma did not progress and it had no effect on recurrence or survival, indicating no clinical significance. And during the same period, The 10-year cumulative recurrence rate of remnant thyroid for unifocal papillary thyroid carcinoma after lobectomy was 8.7%, with no significant difference compared with unilateral multifocal cases (*P* = .866). In addition, only 1 case in this study died of the disease (from a lung metastasis), and the 10-year disease-specific survival rate was more than 96%, indicating an excellent prognosis. So we think that there is no need to perform completion thyroidectomy immediately for unilateral multifocal PTC.

The recurrence-free survival rate in this study was 83.2% and only 14 cases had recurrence and reoperation, including 6 cases for the recurrence in the contralateral remnant thyroid. The recurrence rate of remnant thyroid in patients with ETE was significantly higher than that in patients without ETE (13.2% vs 5.2%, *P* = .044). There were also significant differences in the recurrence rates in the remnant thyroid between different pT stages. The recurrence rate in the remnant thyroid for patients with pT4 stage tumors was significantly higher than that for T1, T2, and T3 tumors (*P* <.0001). However, the recurrence rate of remnant thyroid in patients with coexistent benign nodules in the contralateral lobe was lower than that in patients without contralateral nodules. This might be related to the strategy we used. Contralateral nodules were resected if they were palpated intraoperatively and if contralateral occult carcinoma was diagnosed by frozen section, total thyroidectomy was performed.

This was a retrospective analysis, and the sample size was small because only cases diagnosed as unilateral multifocal carcinoma after lobectomy were included. A total of 32 cases with clinically lymph node metastases (cN1) and 31 cases with pT3/T4 stage were included in this cohort. All of these patients received lobectomy (with isthmectomy) although recent guidelines recommended total thyroidectomy and some of them were also suggested to receive iodine 131 therapy.^[6]^ Even so, only 10.56% (13/123) of all patients required a second surgery resecting the contralateral remnant thyroid.

In our opinion, lobectomy (with isthmectomy) is effective for most patients with unilateral multifocal PTC. ETE was a significant prognostic factor for the recurrence the remnant thyroid and pT stage was an independent prognostic factor for tumor recurrence-free survival. Close follow up is highly recommended for cases with locally progressive tumors (e.g., with ETE or invasion of the central soft tissues). If recurrence or metastases are observed during the follow up, completion thyroidectomy should be done immediately.
